# Acceptability of smartphone applications for global positioning system (GPS) and ecological momentary assessment (EMA) research among sexual minority men

**DOI:** 10.1371/journal.pone.0210240

**Published:** 2019-01-28

**Authors:** Dustin T. Duncan, Su Hyun Park, William C. Goedel, Diana M. Sheehan, Seann D. Regan, Basile Chaix

**Affiliations:** 1 NYU Spatial Epidemiology Lab, Department of Population Health, NYU School of Medicine, New York, New York, United States of America; 2 Department of Epidemiology, Florida International University Robert Stempel College of Public Health and Social Work, Miami, Florida, United States of America; 3 Inserm, Sorbonne Universités, UPMC Univ Paris 06, UMR_S 1136, Pierre Louis Institute of Epidemiology and Public Health, Paris, France; Public Library of Science, UNITED KINGDOM

## Abstract

**Background:**

Emerging research is using global positioning system (GPS) and ecological momentary assessment (EMA) methods among sexual minority men (SMM), a population that experiences multiple health disparities. However, we are not aware of any research that has combined these approaches among SMM, highlighting the need for acceptability and feasibility research. The purpose of this study was to examine the acceptability of implementing GPS and EMA research protocols using smartphone applications among SMM as well as related socio-demographic correlates.

**Methods:**

Data come from a sample of SMM on a popular geosocial-networking app in Paris, France (*n* = 580). We assessed the acceptability of implementing GPS and EMA research protocols on smartphone apps as well as socio-demographic characteristics (i.e., age, sexual orientation, country of origin, employment status, and relationship status). We examined the anticipated acceptability of GPS and EMA data collection methods as well as socio-demographic correlates of acceptability of GPS and EMA methods.

**Results:**

We found that over half (54.1%) of the sample was willing to download a smartphone app for GPS-based research and we found that almost 60% of the participants were willing to download a smartphone app for EMA-based research. In total, 44.0% reported that they were willing to download both GPS and EMA apps. In addition, we found that older participants were less willing to download a smartphone app for EMA research than younger participants aged 18–24 (40–49 years: aPR = 0.40; 95% CI = 0.20, 0.78) and students were more willing to download smartphone apps for both GPS and EMA research (aPR = 1.41; 95% CI = 1.02, 1.95).

**Conclusion:**

Results from this study suggest that using smartphone apps to implement GPS and EMA methods among some SMM are acceptable. However, care should be taken as segments of SMM are less likely to be willing to engage in this type of research.

## Introduction

Ecological momentary assessment (EMA) includes a range of methods aimed at capturing data on health behaviors and outcomes in real-time from participants who self-report as they experience their daily lives [1]. These methods were developed in response to the limitations of self-report retrospective recall, as it can frequently be unreliable and often be systematically biased [2]. These methods recognize that behaviors and experiences are affected by context, so data must be collected on a given experience or behavior in its real-time setting for the data collected to be representative of one’s lived experiences [1]. Studies using these methods often involve repeated measures over varying durations, affording the temporal resolution needed to assess the dynamics of within-subject changes in behavior and experience over time and across contexts [3]. These methods have employed various modalities to collect data on behavior in real-time in real world environments, including emerging research using smartphone applications [4]. These methods of capturing information in real-time are useful tools in studying health behaviors—as they are often discrete and episodic [1]

The contexts of health behaviors, such as the neighborhood environments in which the behaviors are performed, are experienced continuously before, during, and after the performance of the health behavior being studied. [5] As such, integrating EMA methods with global positioning system (GPS) technologies may be an effective strategy for capturing both health behaviors in real time and in their environmental contexts; however, few applications of this combined approach exist in the literature and studies have used different combined GPS-EMA protocols [6, 7]. Epstein and colleagues [6] and Mitchell and colleagues [7], for example, both used a dedicated GPS unit in combination with a separate dedicated personal digital assistant (PDA) for EMA to assess the role of mood and geographic context in substance use. A single smartphone application could have the potential to leverage both a smartphone’s geolocation features to record the device’s latitude and longitude [8] and the ability to deliver prompted questionnaires via the smartphone’s notification system [9].

Importantly, GPS technology is a highly novel approach to studying the neighborhood contexts in which people engage/interact [10]. Indeed, GPS methods allow researchers to examine the multiple spatial contexts that an individual experiences (not just the residential neighborhood environment) and allow researchers to understand the timing and duration of exposures to these spatial contexts (addressing the “uncertain geographic context problem”) [11–13]. Emerging research has employed GPS [14, 15] and EMA methods [16–19] separately among sexual minority men (SMM), a population at high risk for HIV infection and whom experience numerous health disparities [20]. However, no published studies (to the best of our knowledge) have used EMA and GPS approaches simultaneously—a method sometimes referred to as geographically-explicit EMA (GEMA) [6, 21]–among SMM, highlighting the need for feasibility research prior to implementing these methods on larger scales.

As such, the purpose of this study was to examine the anticipated acceptability of using smartphone applications to implement both GPS and EMA research protocols among SMM. In addition, we examined whether socio-demographic characteristics were correlated with a participant’s willingness to download a smartphone application for GPS- and EMA-based research. We hypothesized the combined GPS-EMA research protocols using smartphone applications would be acceptable among SMM, however we also expected there to be socio-demographic variation.

### Methods

#### Sample

We used broadcast advertisements in October 2016 on a popular geosocial-networking smartphone application for SMM to recruit participants. We limited the advertisements to users in the Paris (France) metropolitan area. Users were shown an advertisement with text encouraging them to click through the advertisement to complete an anonymous web-based survey. We provided an incentive to encourage participation, which we described prior to survey administration and implemented after survey administration. In particular, the advertisement described that users who completed the survey were entered in a chance to win €65, which is approximately $70. The advertisement was placed during three consecutive 24-hour weekday periods. After implementing precautions to avoid and eliminate duplicate responses [22], we found no apparent duplicate responses. Our survey included 52 items; the survey was translated from English into French using an adaptation of the TRAPD (translate, review, adjudicate, pretest, document) translation protocol [23] and in this study five French speakers conducted the survey translation. The survey took an average of 11.4 minutes for users to complete. The survey was offered in French and English. Most participants (94.3%), however, took the survey in French. At the end of the recruitment period, 5,206 users had clicked on the advertisement and reached the landing page of the survey, 935 users provided informed consent and began the survey, and 580 users provided informed consent and completed the survey. This represents an overall completion rate of 62% and an overall response rate of 11.1%. All protocols were approved by the New York University School of Medicine Institutional Review Board prior to data collection.

#### Anticipated acceptability of GPS and EMA smartphone-based app research

We asked participants, “Would you download a smartphone app that tracked where you went using global positioning system (GPS) technology for the purposes of a research study?” Response options were “Yes” and “No”. In addition, we asked participants “Would you download a smartphone app that asked you questions throughout the day about your current mood, surroundings, and feelings for the purposes of a research study?” Response options were “Yes” and “No”. For the analysis, we generated binary variables indicating the acceptance of only one of these applications (i.e., accepting of GPS but not EMA and accepting of EMA but not GPS) as well as made a composite variable of willingness to download a GPS and EMA app.

#### Socio-demographic characteristics

We assessed age (categorized into five groups: 18–24 years, 25–29 years, 30–39 years, 40–49 years, 50 years and older), sexual orientation (gay, bisexual, other), whether or not they had been born in France (yes, no), employment status (employed, unemployed, student, retired), and current relationship status (coded as single or in a relationship).

#### Statistical methods

For this study, we decided on the four groups: neither, EMA alone, GPS alone and GPS and EMA because in addition to combined GPS-EMA protocols, it might be useful to know whether there are specific correlates of willingness to use each method alone versus using both simultaneously, which aligns with the goals of the study. We examined the anticipated acceptability of GPS and EMA data collection methods as well as the socio-demographic characteristics associated with such acceptance. First, a descriptive analysis of the study sample was performed. A chi-square or Fisher’s exact test, as appropriate, was used to examine 1) differences in participant characteristics for each outcome, independently, based on acceptability of the GPS and EMA methods (e.g. GPS only vs. all other); and 2) differences in participant characteristics across the four groups (GPS only, EMA only, GPS and EMA and neither GPS nor EMA). Second, using the log-binomial regression model, we assessed adjusted associations to examine the correlates of anticipated acceptability of the GPS and EMA methods. The adjusted prevalence ratio (aPR) and 95% confidence intervals (CIs) were computed. We estimated the prevalence ratio (as opposed to the odds ratio) because the outcomes were relative common and it is well known in epidemiologic studies that odds ratios overestimate the effect in the context of a non-rare outcome [24–26]. Socio-demographic variables, including age, sexual orientation, origin (born in France), employment status, and current relationship status, were adjusted for. All statistical analyses were conducted using Stata 14.0 (StataCorp, College Station, TX, USA).

## Results

### Study participants

The median age was 34 years old (interquartile range [IQR] = 27.0, 42.0), and most participants identified their sexual orientation as either gay (84.0%) or bisexual (11.9%) (Table 1). Most participants (77.6%) were born in France. Furthermore, most respondents were employed (66.9%), and 14.0% were students. The majority of participants reported being single (65.2%).

**Table 1:** Socio-demographic characteristics of participants according to their willingness to download different type of smartphone applications (N = 580).

	Total, N(%)	GPS Alone	EMA Alone	GPS and EMA	Neither GPS nor EMA	
		n(%)	n(%)	n(%)	n(%)	*p*^a^
Overall	580 (100)	59 (10.2)	82 (14.1)	255 (44.0)	166 (28.6)	
**Age**						0.039
18–24	84 (14.5)	4 (4.8)	22 (26.2)	26 (31.0)	32 (38.1)	
25–29	103 (17.8)	12 (11.7)	15 (14.6)	49 (47.6)	26 (25.2)	
30–39	180 (31.0)	21 (11.7)	21 (11.7)	87 (48.3)	51 (28.3)	
40–49	139 (24.0)	17 (12.2)	18 (13.0)	62 (44.6)	42 (30.2)	
≥ 50	54 (9.3)	5 (9.3)	6 (11.1)	30 (55.6)	13 (24.1)	
**Sexual orientation**						0.925
Gay	487 (84.0)	52 (10.7)	71 (14.6)	220 (45.2)	140 (28.8)	
Bisexual	69 (11.9)	6 (8.7)	10 (14.5)	30 (43.5)	22 (31.9)	
**Born in France**						0.109
Yes	450 (77.6)	40 (8.9)	67 (14.9)	208 (46.2)	134 (29.8)	
No	113 (19.5)	19 (16.8)	15 (13.3)	47 (41.6)	32 (28.3)	
**Employment status**						0.079
Employed	388 (66.9)	45 (11.6)	46 (11.9)	178 (45.9)	118 (30.4)	
Unemployed	84 (14.5)	4 (4.8)	19 (22.6)	38 (45.2)	23 (27.4)	
Student	81 (14.0)	7 (8.6)	16 (19.8)	34 (42.0)	24 (29.6)	
Retired	7 (1.2)	2 (28.6)	1 (14.3)	4 (57.1)	0 (0.0)	
**Current relationship status**						0.964
Single	378 (65.2)	39 (10.3)	55 (14.6)	170 (45.0)	113 (29.0)	
Relationship with a man	172 (29.7)	18 (10.5)	25 (14.5)	81 (47.1)	48 (27.9)	

GPS = global positioning system; EMA = ecological momentary assessment

Values are n(%)

^a^Chi-square test or Fisher’s exact test was used to compare between 4 groups.

#### Acceptability of GPS and EMA smartphone app-based research

A total of 18 people did not answer either the GPS question or EMA question: 17 people were missing responses to both questions and 1 participant did not provide a response for the EMA question. In our analytical sample, more than half (54.1%) the sample was willing to download a smartphone-based GPS application for research purposes. Almost 60% of participants were willing to download a smartphone-based EMA application. In total, 44.0% reported that they were willing to download both the GPS and EMA applications; 10.2% were willing to download the GPS but not the EMA application; 14.1% were willing to download the EMA but not the GPS application; and 166 people (28.6%) were not willing to download either the GPS or the EMA application for research purposes. The tetrachoric correlation between these two dichotomous outcomes (i.e., downloading GPS and downloading EMA) was computed (r = 0.6978).

### Socio-demographic correlates of acceptability of GPS and EMA smartphone app-based research

Individuals born outside France were more willing than those born in France to download an app for a GPS protocol but not for an EMA protocol, whereas unemployed participants were more likely to download an app for an EMA protocol but not for a GPS protocol (also found in our multivariate models described below). Differences were found in the ages of participants according to their willingness to download both GPS and EMA applications (Table 1). In addition, the age distribution across the four groups varied according to individual characteristics (Table 1). Multivariate analyses revealed that, after adjusting for sociodemographic characteristics, older individuals were less willing than the youngest group to download an app for an EMA protocol but not for a GPS protocol (Table 2). For example, compared to those aged 18–24 years, participants aged 40–49 years had an aPR of 0.40 (95% CI = 0.20, 0.78) for downloading an app for an EMA protocol but not for a GPS protocol. Unemployed participants were more willing to download an app for an EMA protocol but not for a GPS protocol than employed participants (aPR = 1.82; 95% CI = 1.11, 2.99). No such relationship was found for the anticipated willingness to download an app for GPS methods but not an app for EMA methods. There was an increasing probability to accept an app for both purposes with increasing age. For example, participants aged 50 years and older were more willing than the youngest group (aPR = 2.24; 95% CI = 1.37, 3.66), but for a given age, students were more likely to accept the app than non-student (aPR = 1.41; 95% CI = 1.02, 1.95). No differences were found in sexual orientation and current relationship status in terms of anticipated acceptability of GPS and EMA protocols.

**Table 2:** Adjusted prevalence ratio (aPR)^a^ and 95% CI of socio-demographic factors associated with SMM’s willingness to download smartphone apps for GPS and EMA methods.

	GPS Alone	EMA Alone	GPS and EMA	Neither GPS nor EMA
	aPR(95% CI)	aPR(95% CI)	aPR(95% CI)	aPR (95% CI)
**Age**				
18–24	Referent	Referent	Referent	Referent
25–29	2.71 (0.82, 8.91)	0.44 (0.23, 0.88)*	2.02 (1.33, 3.07)**	0.54 (0.33, 0.87)*
30–39	2.22 (0.63, 7.82)	0.37 (0.20, 0.71)**	2.12 (1.38, 3.27)**	0.60 (0.38, 0.92)*
40–49	2.65 (0.75, 9.40)	0.40 (0.20, 0.78)**	1.97 (1.25, 3.09)**	0.62 (0.39, 0.98)*
≥ 50	1.10 (0.18, 6.63)	0.39 (0.15, 1.01)	2.24 (1.37, 3.66)**	0.65 (0.36, 1.16)
**Sexual orientation**				
Gay	1.38 (0.52, 3.68)	1.25 (0.63, 2.46)	0.94 (0.71, 1.24)	0.90 (0.61, 1.33)
Bisexual	Referent	Referent	Referent	Referent
**Born in France**				
Yes	0.58 (0.34, 0.99)*	0.98 (0.58, 1.68)	1.20 (0.94, 1.54)	1.00 (0.71, 1.40)
No	Referent	Referent	Referent	Referent
**Employment status**				
Employed	Referent	Referent	Referent	Referent
Unemployed	0.36 (0.11, 1.13)	1.82 (1.11, 2.99)*	0.98 (0.75, 1.28)	0.98 (0.67, 1.43)
Student	1.04 (0.41, 2.66)	0.90 (0.47, 1.74)	1.41 (1.02, 1.95)*	0.68 (0.42, 1.10)
Retired	5.02 (0.85, 29.45)	1.36 (0.18, 10.20)	1.18 (0.58, 2.40)	--
**Current relationship status**				
Single	Referent	Referent	Referent	Referent
Relationship with a man	0.96 (0.56, 1.65)	1.10 (0.70, 1.71)	1.01 (0.82, 1.22)	0.96 (0.72, 1.28)

GPS = global positioning system; EMA = ecological momentary assessment; CI = confidence interval

^a^Adjusted for age, sexual orientation, origin (born in France), employment status, and current relationship status

*p<0.05;

**p<0.01

## Discussion

The purpose of this study was to examine the anticipated acceptability of SMM downloading smartphone applications for GPS and EMA protocols. As previously discussed, emerging GPS and EMA research has been conducted among SMM, however, to our best knowledge, no study has *combined* GPS and EMA methods among any SMM population. In addition, none of the existing GPS studies have used a smartphone app for data collection in a SMM population. But rather, existing research has utilized a dedicated GPS device [14, 15]. A recent study that evaluated the anticipated acceptability of text message- and voice-based EMA methods among a sample of 74 young SMM found that almost all participants (96%) reported that they would be willing to accept texts on their smartphone to answer questions about their current mood, surroundings, or feelings [27]. In addition, a very large majority (89%) reported being willing to accept phone calls to answer these questions [27]. Among existing studies that implemented EMA methods among SMM, two studies have used smartphone applications on a non-participant smartphone [17, 18] and one allowed participants to use their own smartphones to participate [19]. Willingness to download an app to a personal phone may increase compliance to research protocols.

We recognize that use of an EMA application increase cellular data; however, use of an EMA application as compared to EMA texts might be more feasible in population-based research health research, in part because participants can be charged for text messages—making them less likely to engage in research protocols that involve texts being sent to them. In addition, apps might provide more flexibility for the researcher in terms of questions, responses and other special features and apps may be more visually appealing and consequently encourage participation in health-related research. In this study, willing to downloading a smartphone app for GPS methods alone and downloading a smartphone app for EMA protocols alone were moderately correlated and we found that most SMM were willing to download a smartphone app for either purpose. However, there was socio-demographic variation, where, for example, older participants were more willing to download a smartphone application for both GPS and EMA protocols.

The variation in willingness to download these apps by age is interesting: the youngest are more reluctant to use an app for GPS-based research but are more open to using an app for EMA-based research, underlining that these two approaches have different implications in terms of privacy. Perhaps, there is a stronger feeling of distrust among out-of-school young SMM. Future studies could examine concerns related to the use of smartphone apps for GPS and EMA protocols with SMM using qualitative methods such as semi-structured interviews. Future studies could also test the feasibility of implementing GPS and EMA studies where SMM download smartphone apps to track their mobility and examine their health behaviors in real-time, including with large samples of SMM across geographies as well as assessing the feasiblity of collecting GPS and EMA data in SMM populations for different research topics (e.g., research on sexual behavior, drug use and/or mental health), which may impact feasibility. These studies would advance the field substantially by providing a nuanced spatio-temporal link between environment and risk behavior, also because there may be differences in hypotheticals for engagement in a research study versus real life behaviors in this particular population. This future research on neighborhoods and the health of gay, bisexual and other SMM, is warranted in light of increasing research demonstrating that neighborhoods can influence HIV outcomes in SMM populations [28] as well as other health outcomes/behaviors [29, 30]. The implications for this next-generation work are that they can potentially revolutionize our researchers and practitioners’ views of biomedical prevention, including pre-exposure prophylaxis (PrEP) interventions.

## Limitations

This study has limitations. Social desirability bias is possible. In addition, this study was conducted in a single urban geographic location in Western Europe among a sample of SMM recruited from a single geosocial-networking application, which limits the generalizability of our findings. For example, SMM who use geosocial-networking applications might be different from those who do not. In particular, SMM who use geosocial-networking applications may be more willing than SMM who do not download other applications, including those for GPS tracking, as these geosocial-networking applications already utilize a smartphone’s geolocation features to connect their users with other nearby users [31]. Furthermore, these data were collected two years ago and the expansion of smartphones and attitudes toward technology may have changed since then.

## Conclusion

We found that over half (54.1%) of the sample was willing to download a smartphone app for GPS-based research and almost 60% of the participants were willing to download a smartphone app for research using EMA methods. We also found socio-demographic variation in willingness to download these apps. Therefore, care should be taken when conducting this research—as segments of SMM are less likely to be willing to engage in this type of research. Future studies using GPS and EMA methods together will advance the field substantially by providing a nuanced spatio-temporal link between environment and risk behavior in SMM, a population that experiences multiple health disparities.
